# Awareness, Registration Status, and Perceived Barriers to Ayushman Bharat Health Account (ABHA) Adoption Among Patients in Jharkhand: A Cross-Sectional Study

**DOI:** 10.7759/cureus.117541

**Published:** 2026-10-06

**Authors:** Esha Sharan, Bhoopendra Singh, Shashi B Singh

**Affiliations:** 1 Hospital Administration, Rajendra Institute of Medical Sciences, Ranchi, IND; 2 Forensic Medicine and Toxicology, Rajendra Institute of Medical Sciences, Ranchi, IND; 3 Community Medicine, Rajendra Institute of Medical Sciences, Ranchi, IND

**Keywords:** abdm, abha, ayushman bharat health account, digital health, electronic health records, health infrastructure, health literacy, public health

## Abstract

Background: The Ayushman Bharat Health Account (ABHA), introduced under the Ayushman Bharat Digital Mission (ABDM), is intended to facilitate the digital management and exchange of health records in India. However, awareness of ABHA, knowledge of its registration pathway, and actual account creation among patients remain uncertain. This study assessed ABHA awareness and registration-related knowledge among adult outpatients at a tertiary care teaching hospital in Jharkhand, determined the proportion reporting personal ABHA account creation, and examined attitudes, perceived barriers, awareness-to-registration gaps, and their associations with selected sociodemographic characteristics.

Methods: A cross-sectional study was conducted between May and July 2026 among outpatients at Rajendra Institute of Medical Sciences (RIMS), Ranchi, Jharkhand, a 2,327-bed government tertiary care teaching hospital with a daily outpatient department (OPD) footfall of 2,100-3,000 patients. A convenience sample of 400 adult patients (aged ≥20 years) was surveyed. Participants completed a pre-structured bilingual questionnaire with sociodemographic information, baseline awareness, registration status, and barriers to registration. Chi-square tests were used to find the relationship between sociodemographic variables and awareness levels, with statistical significance set at p<0.05.

Results: Among 400 participants, 202 (50.5%) were male. Overall, 187 (46.8%) had heard of ABHA, 64 (16.0%) knew accounts could be created online or via an application, and only 24 (6.0%) had personally created an account. The mean composite knowledge score was 7.34±2.75 (56.5% of maximum), while high perceived barriers (≥75% score) were reported by 295 (73.8%) participants. Composite knowledge was significantly associated with age (χ^2^(4)=15.795, p=0.003; linear trend M^2^=10.61, p=0.001), with older adults exhibiting lower knowledge. Gender showed a nominal association (χ^2^(2)=7.403, p=0.025) that did not survive Bonferroni correction (p<0.003125). Education (χ^2^(6)=4.854,p=0.563), socioeconomic status (χ^2^(6)=7.399, p=0.286), occupation, residence, religion, and PM-JAY status showed no significant association with knowledge or perceived barrier levels.

Conclusion: Low ABHA registration (6.0%) in this tertiary care population was accompanied by a pronounced awareness-to-action gap, with the steepest attrition occurring at knowledge of the self-registration route (16.0%) rather than general awareness (46.8%). Pervasive perceived barriers (73.8%) cut uniformly across demographic lines. To bridge this gap, hospital administration should transition from passive promotion to active, assisted on-site registration desks in OPDs, prioritising older outpatients.

## Introduction

Digital health has moved from the periphery of health system reform to its centre. Interoperable records, consent-based data exchange and remote consultation are now treated as prerequisites for universal health coverage rather than optional add-ons, and India's own reform debate has framed the shift in explicitly citizen-centric terms [1]. The appeal is practical: when a patient’s history travels with them, clinicians repeat fewer investigations, referrals lose less information, and continuity of care survives the move from one facility to another. In low-resource settings, however, systems built around smartphone ownership and stable connectivity risk widening the very gaps they were meant to close [2].

India has invested heavily in financial protection through the Pradhan Mantri Jan Arogya Yojana (PM-JAY), which extended hospitalisation cover to a large share of the population [3]. Coverage alone, however, has not resolved the underlying information problem. Records remain fragmented across facilities, diagnostic tests are duplicated, and claims processing is slowed by paper-based verification. Studies of PM-JAY beneficiaries have repeatedly found that awareness of an entitlement and actual use of it are two different things, with utilisation lagging well behind enrolment even in districts where the scheme is well-publicised [4].

The Ayushman Bharat Digital Mission (ABDM), launched in September 2021, was the government's response to that gap. Its central instrument is the Ayushman Bharat Health Account (ABHA), a 14-digit identifier that allows a person to link and share health records across participating facilities under their own consent.

Progress on registration has been rapid on paper: an assessment of the mission using a health system control-knob framework recorded more than 400 million beneficiary registrations and over 270 million linked health records within roughly two years of launch, alongside verification of more than 208,000 health facilities [5]. Such an identifier layer is also what makes population-scale analytics, telemedicine and longitudinal surveillance technically feasible [6].

Registration counts, however, are a weak proxy for actual adoption, as evidence across diverse Indian settings consistently reveals sharp drop-offs between macro-level enrolment targets and patient-level operational uptake. Work from Chandigarh conducted during the pilot phase of the mission found that although digital health ID uptake reached 78% where enrolment was actively facilitated at a primary health centre, only 28.2% of the same participants had ever heard of electronic health records and only 35.6% had heard of ABDM, with lack of awareness, digital-literacy apprehension, and perceived low utility emerging as the dominant barriers [7]. A hospital-based survey in urban Bengaluru reported that just 8% of outpatients knew of ABDM despite high familiarity with other government digital services [8]. Among underprivileged communities in Bhopal and Raisen districts, registration remained in the low single digits, with absent Aadhaar numbers (linked to India’s government-issued biometric national ID system) and unlinked mobile numbers acting as hard constraints [9]. A prospective audit of ABHA integration at a tertiary centre in Eastern India traced failures to a narrow set of operational hurdles, chiefly absence of a smartphone, poor signal reception and outright refusal [10]. Taken together, these findings suggest that the binding constraints sit downstream of awareness campaigns, in the mechanics of enrolment itself.

Who is left behind matters as much as how many. Digital health inequalities cluster along age, gender, income and literacy lines, and interventions that assume self-directed registration through a mobile application tend to reproduce those inequalities rather than correct them [11]. Eastern India is a relevant test case. Rural and tribal populations in the region report low healthcare utilisation, high out-of-pocket expenditure, and continued reliance on informal providers, and awareness of even well-established Ayushman Bharat entitlements has been shown to be uneven across rural communities in the region [12]. Jharkhand shares these characteristics, yet patient-level evidence on ABHA from the state is essentially absent. National dashboards track ABHA generation; they do not tell a hospital administrator why the patient sitting in the OPD queue has not created one.

The study was therefore undertaken at a large public tertiary care hospital in Ranchi to measure what outpatients actually know about ABHA, how many have created an account, what they believe about digital health records, and which specific obstacles they report. The intention is to identify the points in the enrolment pathway where hospital-level and state-level action would make the most difference.

We therefore conducted a cross-sectional study at a large public tertiary care hospital in Ranchi. The primary objective was to assess ABHA awareness and knowledge of its registration pathway among adults aged ≥20 years attending the outpatient department of a tertiary care teaching hospital in Jharkhand, and to determine the proportion who reported having personally created an ABHA account during the study period.

Secondary objectives were (i) to describe participants’ attitudes toward ABHA and the perceived barriers to registration, based on responses to the study questionnaire; (ii) to identify specific knowledge gaps across the sequential steps from ABHA awareness to registration; (iii) to examine the association of composite knowledge and perceived-barrier levels with selected sociodemographic characteristics, including age, gender, education, socioeconomic status, occupation, residence, religion, and PM-JAY beneficiary status.

## Materials and methods

Study design and setting

A hospital-based, descriptive cross-sectional study was carried out in the outpatient departments of Rajendra Institute of Medical Sciences (RIMS), Ranchi, Jharkhand, India. RIMS is the state's principal government tertiary care teaching hospital and its main referral centre, with an average daily OPD attendance of approximately 2,100 to 3,000 patients [13]. The study assessed patient awareness, conceptual understanding of ABHA, self-reported registration status, attitudes and operational obstacles to registration. Reporting follows the Strengthening the Reporting of Observational Studies in Epidemiology (STROBE) statement for cross-sectional studies; the completed checklist is provided in Appendix 1 [14].

Study population and eligibility criteria

The study population comprised adult patients attending general and speciality OPD clinics during the data collection period. Adults aged 20 years and above who were able to communicate in Hindi or English and who provided written informed consent were eligible. Patients presenting with acute medical emergencies, those with cognitive or communication impairment sufficient to prevent completion of the interview, and those who declined participation were excluded.

Sample size and sampling technique

Sample size was estimated using the standard formula for a single proportion, assuming a baseline ABHA awareness prevalence of 50% to yield the maximum requirement, an absolute margin of error of 7%, and a 95% confidence level (Z = 1.96) [15].

$n = \frac{Z^2 P(1-P)}{d^2}$

This yielded 196 participants, rising to 218 after a 10% allowance for non-response. To improve precision within demographic subgroups and to permit stable cross-tabulation, the target was expanded to 400. Eligible respondents were recruited by convenience sampling in OPD waiting areas during clinic hours. Recruitment took place from Monday to Saturday between 9:00 am and 1:00 pm and 2:00 pm and 5:00 pm on Monday to Friday, and between 9:00 am and 2:00 pm on Saturday, in the outpatient waiting areas of the hospital. Of the approximately 2,100 to 3,000 patients approached during the study period, 400 who met the eligibility criteria willingly participated in the interview. Because sampling was non-probabilistic, selection bias cannot be excluded.

Study instrument and domain structure

Data were collected using a self-designed and structured, pre-tested bilingual (English and Hindi) questionnaire. Content validity was reviewed by public health faculty, and the instrument was pilot-tested on 40 OPD patients whose responses were excluded from the final analysis. The tool covered four sections. In the study sample, internal consistency was evaluated using Cronbach's α, yielding α = 0.71 for the 13-item knowledge composite (indicating acceptable reliability) and α = 0.26 for the five-item barrier composite (indicating low internal consistency, reflecting the heterogeneous nature of individual, technical, and institutional obstacles). These values are reported as observed, and the modest reliability of the barrier composite is explicitly noted as a study limitation. Test-retest reliability was not assessed.

The first section recorded sociodemographic details: age group, gender, education, occupation, religion, place of residence, PM-JAY beneficiary status, and socioeconomic status classified using the modified Kuppuswamy scale updated for 2024 using the Consumer Price Index (CPI) base year adjustments [16].

The second section comprised 13 knowledge/awareness items covering recognition of ABHA (Q1), digital storage of health records (Q2), use of Aadhaar for ABHA creation (Q3), registration through a mobile app or online portal (Q4), free registration (Q5), linking past health records (Q6), a participant's own ABHA creation status (Q7, analysed separately), awareness that an ABHA number can be created through an online portal/app (Q8), possible internet requirements for registration (Q9), mobile-number linkage (Q10), awareness of the statement that Aadhaar is mandatory for registration (Q11), use across multiple hospitals (Q12), online access to laboratory reports, prescriptions, and medical history (Q13), and distinction between ABHA and the PM-JAY insurance card (Q14). Q4 and Q8 are closely overlapping items in the questionnaire, and this redundancy is considered in the limitations. The survey instrument was structured into distinct analytical domains to differentiate baseline recognition from procedural comprehension. Although items Q4 and Q8 featured identical statement phrasing regarding online/mobile application registration, they were intended to capture different levels of understanding: Q8 was interpreted as recognition that online/app registration routes exist, and Q4 as knowledge that registration can be done through them. Both items contributed to the knowledge composite.

The third section captured the behavioural indicator of personal ABHA account creation status (Q7), which was analysed separately and excluded from the composite knowledge score. The fourth section contained 10 items on attitudes, barriers, and support needs (Q15 to Q24). Items Q15 to Q19 were descriptive. Items Q20 to Q23 assessed perceived enrolment obstacles (information lack, technical/OTP failures, device/internet limitations, and privacy concerns), while Q24 assessed whether awareness drives and staff assistance would improve registration. Because Q24 measures institutional support needs and facilitators rather than a pure obstacle, the five-item composite (Q20-Q24) is interpreted as a combined perceived difficulty/support score rather than a pure barrier scale.

Empirical validation confirmed that retaining Q24 improved scale internal consistency (α = 0.26 vs. α = 0.18 without Q24) and yielded 90% concordance in participant risk classification. All five items were evaluated individually and as a composite score. The detailed questionnaire is attached in Appendix 2.

Scoring and classification

A single scoring rule was applied to both composite scales so that the two are directly comparable: a response indicating knowledge or endorsing a barrier scored 1 point, "Not sure" scored 0.5 points, and a negative response scored 0. The knowledge composite therefore ranges from 0 to 13 and the barrier composite from 0 to 5. Item Q11, which asks about awareness of the statement that Aadhaar is mandatory for ABHA registration, is negatively/ambiguously worded relative to the other knowledge items: current National Health Authority procedures permit ABHA generation through alternative identity documents as well as Aadhaar, so an affirmative response is not unambiguously factually correct. The item was nonetheless scored affirmative-as-correct, since it was written to capture the widely held perception that Aadhaar is required, rather than to test knowledge of the verified procedural fact; its influence on the composite score is examined in a sensitivity analysis excluding this item. Each was converted to a percentage of the maximum attainable score and classified using near-identical cut-points: low (<50%), moderate (50.0% to 74.9%) and high (≥75%).

This scoring rule (Yes = 1, Not sure = 0.5, No = 0) was applied consistently to all knowledge and barrier items and was the only scoring rule used in this study; no alternative scoring rule was analysed. 

Data collection

Face-to-face interviews were conducted by a single trained investigator who conducted all 400 interviews in English or Hindi between May and July 2026. Interviews took place in semi-private seating areas within OPD lounges to protect participant privacy, and each session lasted approximately 10 to 15 minutes. Written informed consent was obtained before administration. Completed questionnaires were checked daily for completeness.

Statistical analysis

Data were independently double-entered into Microsoft Excel by investigators (Microsoft Corp., Redmond, WA). Discrepancies between entry sheets were identified using automated cell comparison formulas and resolved by cross-checking against the original paper questionnaires. Statistical analysis was performed using Jamovi version 2.7 (https://www.jamovi.org). Categorical variables and item-level responses were summarised as frequencies and percentages, and composite scores as mean ± standard deviation with median and range. Associations between sociodemographic variables and composite knowledge and barrier levels were tested using Pearson's chi-square test, with Cramér's V reported as the effect size. Because age is an ordinal variable, its association with knowledge was additionally examined using a linear-by-linear association (trend) test.

To satisfy Cochran's rule that no more than 20% of cells should have an expected count below 5, three categories were collapsed before hypothesis testing: participants aged 20 to 29 years (n = 11) were combined with those aged 30 to 39 years; the single Jain respondent was combined with the "Other" religion category; and the upper and upper-middle socioeconomic classes were merged. For the same reason, the barrier outcome was dichotomised as high versus low or moderate for hypothesis testing, since only 12 participants fell into the low band. The three participants who were unsure of their PM-JAY status were reported descriptively but excluded from the PM-JAY hypothesis test. After these adjustments, no test violated Cochran's rule.

A two-tailed p-value below 0.05 was considered statistically significant for individual comparisons. Because 16 chi-square tests were performed across two outcomes and eight exposures, a Bonferroni-corrected threshold of p < 0.003125 is also reported, and results that meet the nominal but not the corrected threshold are identified as such. All analyses were unadjusted; no multivariable modelling was undertaken.

Ethical considerations

The protocol was approved by the Institutional Ethics Committee of Rajendra Institute of Medical Sciences, Ranchi (approval letter no. 53 with synopsis submission no. S/47/26, dated February 21, 2026), before the start of data collection. The study adhered to the Declaration of Helsinki and to the national ethical guidelines for biomedical and health research involving human participants. Confidentiality was maintained by assigning unique numerical codes to all records, and no personal identifiers were retained in the analysis dataset.

## Results

Participant characteristics

All 400 participants completed the interview, with no missing responses. Men and women were almost equally represented (n=202; 50.5% vs. n=198; 49.5%). The largest age group was 30-39 years (n=184; 46.0%), followed by ≥50 years (n=110; 27.5%) and 40-49 years (n=95; 23.8%); only 11 (2.8%) were aged 20-29. Education levels varied: 34.0% (n=136) had no formal schooling, 25.2% (n=101) primary, 32.0% (n=128) secondary, and 8.8% (n=35) higher secondary. On the modified Kuppuswamy scale, most belonged to the lower-middle (36.5%; n=146) or middle (30.0%; n=120) classes, with 19.5% (n=78) in the lower class. Nearly half lived in urban areas (n=195; 48.8%), and 43.2% (n=173) reported being PM-JAY beneficiaries. The full profile appears in Table 1.

**Table 1:** Sociodemographic profile of study participants (N = 400) Sociodemographic characteristics of the 400 outpatients surveyed at Rajendra Institute of Medical Sciences, Ranchi. PM-JAY, Pradhan Mantri Jan Arogya Yojana. Socioeconomic status was classified using the modified Kuppuswamy scale [16]. Categories are presented as recorded; three of them were collapsed before hypothesis testing as described in the Methods. Percentages are calculated on the full sample and may not sum to 100.0 because of rounding. There were no missing data for any variable.

Variable	Category	N (%)
Gender	Male	202 (50.5)
	Female	198 (49.5)
Age group (years)	20-29	11 (2.8)
	30-39	184 (46.0)
	40-49	95 (23.8)
	50 and above	110 (27.5)
Education	No formal schooling / other	136 (34.0)
	Primary	101 (25.2)
	Secondary	128 (32.0)
	Higher secondary	35 (8.8)
Religion	Hindu	164 (41.0)
	Muslim	124 (31.0)
	Other	57 (14.2)
	Christian	54 (13.5)
	Jain	1 (0.2)
Socioeconomic status	Upper	35 (8.8)
	Upper-middle	21 (5.2)
	Middle	120 (30.0)
	Lower-middle	146 (36.5)
	Lower	78 (19.5)
Occupation	Employed	103 (25.8)
	Student	88 (22.0)
	Unemployed	82 (20.5)
	Self-employed	64 (16.0)
	Homemaker	63 (15.8)
Residence	Urban	195 (48.8)
	Rural	158 (39.5)
	Semi-urban	47 (11.8)
PM-JAY beneficiary	Yes	173 (43.2)
	No	224 (56.0)
	Not sure	3 (0.8)

The awareness-to-action gap

The three indicators that mark successive stages of the enrolment pathway, namely having heard of ABHA (Q1), knowing that an account can be created independently online or through a mobile application (Q4), and having actually created one (Q7), are shown in Figure 1. Attrition across the pathway was steep and was concentrated at the second step: the fall between hearing of ABHA and knowing how to obtain one was roughly three times larger than the fall between knowing how and doing it. Awareness of the self-registration route was the single lowest-scoring item in the entire knowledge domain, and more than three-quarters of the sample answered it in the negative. A further 40 participants were unsure whether they held an account, so even the modest registration figure carries some uncertainty (Figure 1). 

**Figure 1:**
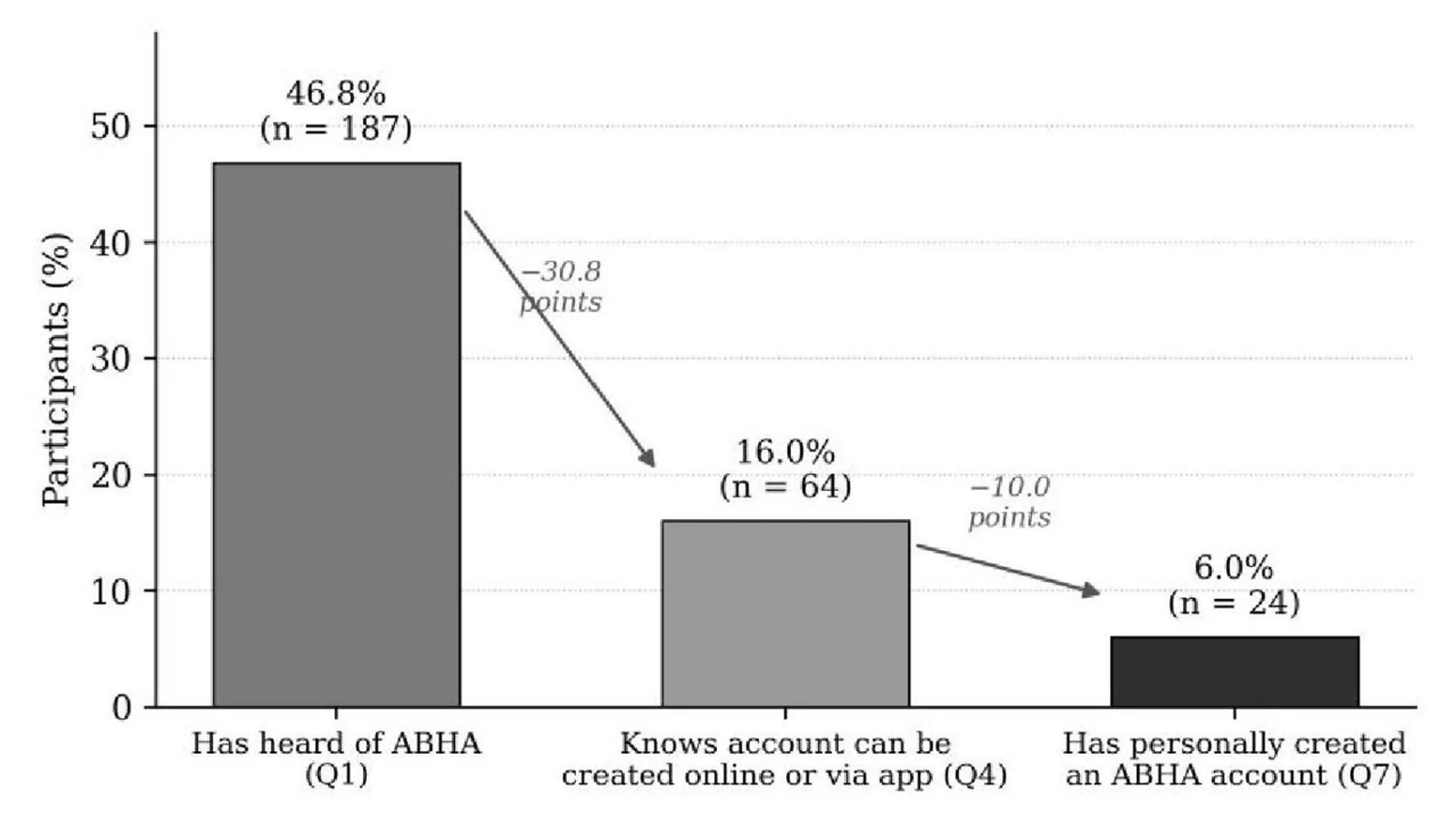
Awareness-to-action gap in ABHA adoption Awareness-to-action gap in ABHA adoption (N = 400). Bars show the proportion of participants who reported having heard of ABHA (item Q1), who knew that an ABHA account can be created independently through an online portal or mobile application (item Q4), and who had personally created an account (item Q7). Arrows indicate the absolute percentage-point fall between consecutive stages. Values above bars give the percentage and the corresponding number of participants. ABHA, Ayushman Bharat Health Account.

Item-level knowledge

Responses to the remaining 11 knowledge items are given in Table 2. Roughly half the sample endorsed each of the structural features of ABHA, with the proportion answering affirmatively ranging from just under half to a little over half across every item. Familiarity was highest for the practical prerequisites of enrolment, namely the need for a linked mobile number and the existence of a dedicated mobile application, and lowest for the conceptual distinction between ABHA and the PM-JAY insurance scheme, which fewer than half the participants identified correctly and about which a further sixth were unsure. Uncertainty rather than outright denial accounted for a substantial share of non-affirmative responses throughout, with the "Not sure" option selected by between an eighth and a sixth of participants on most items (Table 2). 

**Table 2:** Item-wise responses to the composite knowledge items (N = 400) Distribution of responses to 11 of the 13 items forming the composite knowledge score. Items Q1 and Q4, which also contribute to the composite, are presented in Figure 1 alongside the behavioural indicator Q7 and are therefore not repeated here. Item Q7 was analysed as an independent behavioural indicator and did not contribute to the composite score. Item content is abbreviated; the full bilingual wording is available from the corresponding author. ABHA, Ayushman Bharat Health Account; PM-JAY, Pradhan Mantri Jan Arogya Yojana.

Item	Content (abbreviated)	Yes, n (%)	No, n (%)	Not sure, n (%)
Q2	ABHA stores health records digitally	198 (49.5)	150 (37.5)	52 (13.0)
Q3	ABHA number can be generated using Aadhaar card	220 (55.0)	123 (30.8)	57 (14.2)
Q5	ABHA creation is free of cost	215 (53.8)	134 (33.5)	51 (12.8)
Q6	ABHA helps link past health records for future access	208 (52.0)	137 (34.2)	55 (13.8)
Q8	ABHA number can be registered at an online portal/app	221 (55.2)	114 (28.5)	65 (16.2)
Q9	Internet connection may be required for ABHA registration	217 (54.2)	117 (29.2)	66 (16.5)
Q10	ABHA number can be linked to a mobile number	224 (56.0)	111 (27.8)	65 (16.2)
Q11	Aadhaar card is mandatory for ABHA registration	208 (52.0)	142 (35.5)	50 (12.5)
Q12	ABHA number can be used in multiple hospitals	212 (53.0)	130 (32.5)	58 (14.5)
Q13	Lab reports and prescriptions are available online	200 (50.0)	132 (33.0)	68 (17.0)
Q14	ABHA is distinct from PM-JAY insurance	191 (47.8)	138 (34.5)	71 (17.8)

Attitudes toward digital health records were consistently favourable and were more strongly endorsed than any knowledge item (Table 3). Roughly three-quarters of participants agreed that digital records would improve the quality of their care, said they would encourage family members to register, and expected ABHA to reduce repeated paperwork and waiting time. The strongest single attitude recorded in the survey concerned institutional support: nearly four in five said that a dedicated helpdesk or registration kiosk within the hospital would make them more likely to enrol. Expressed trust that health data would be kept secure was also high, a point that sits in tension with the barrier findings reported below (Table 3). 

**Table 3:** Distribution of responses to the five descriptive attitude and support-preference items These items were not scored and did not contribute to either composite score. The five enrolment-related obstacle/support items (Q20 to Q24) form the combined difficulty/support composite and are presented in Figure 4. ABHA, Ayushman Bharat Health Account

Item	Content (abbreviated)	Yes, n (%)	No, n (%)	Not sure, n (%)
Q15	ABHA is a useful initiative for improving healthcare services	302 (75.5)	55 (13.8)	43 (10.8)
Q16	Would encourage family members to register	303 (75.8)	50 (12.5)	47 (11.8)
Q17	Hospital helpdesk or kiosk would increase enrolment	318 (79.5)	50 (12.5)	32 (8.0)
Q18	ABHA would reduce paperwork and waiting time	310 (77.5)	56 (14.0)	34 (8.5)
Q19	Trusts that health data would be kept secure	308 (77.0)	40 (10.0)	52 (13.0)

Composite knowledge and barrier levels

Participants achieved slightly more than half of the maximum possible knowledge score, indicating an overall moderate level of knowledge, with considerable variability among respondents. In contrast, the high mean barrier score indicated that participants perceived considerable barriers, although the extent of these barriers varied across individuals. The descriptive statistics for the composite knowledge and barrier scores are presented in Table 4. 

**Table 4:** Distribution of composite knowledge and barrier scores among participants Summary statistics of composite knowledge and perceived barrier scores regarding ABHA among study participants (N = 400). ABHA, Ayushman Bharat Health Account

Score Domain	Mean ± SD	Median	Range	Maximum Possible Score	Mean as % of Maximum
Knowledge score	7.34 ± 2.75	7.5	1.0-13.0	13	56.5%
Perceived barrier score	4.10 ± 0.88	4.10	0.0-5.0	5	82.0%

The two distributions are contrasted in Figure 2, and their shapes are markedly different. Knowledge was spread across all three bands with the largest share in the middle, whereas barriers were heavily concentrated in the highest band, with only a small minority of participants reporting few obstacles. In other words, what participants knew varied considerably from person to person, while what stood in their way did not (Figure 2). 

**Figure 2:**
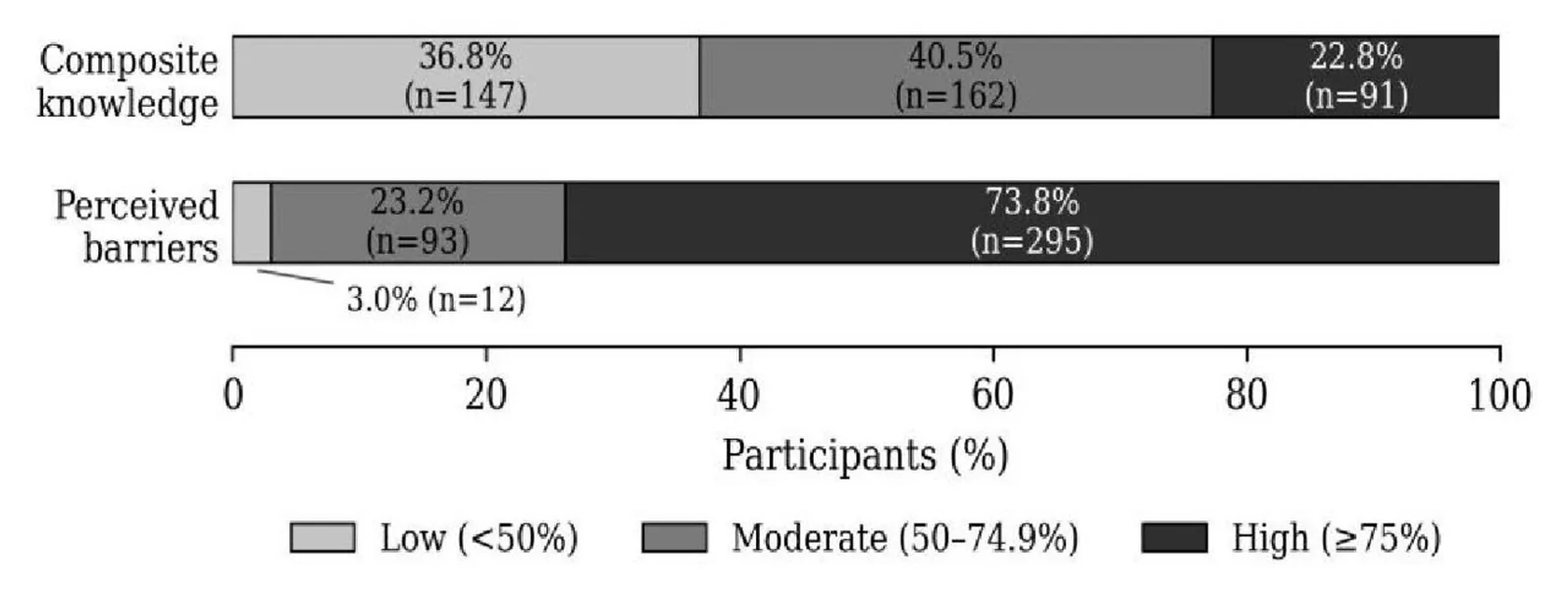
Distribution of composite knowledge and perceived-barrier levels Both composites were scored using the same rule (yes = 1, not sure = 0.5, no = 0), expressed as a percentage of the maximum attainable score (13 points for knowledge, 5 points for barriers) and banded using identical cut-points: low (<50%), moderate (50.0-74.9%) and high (≥75%). Segment labels give the percentage and number of participants in each band.

Sociodemographic associations with knowledge

Two of the eight sociodemographic variables were associated with composite knowledge level, and both are shown in Figure 3. Age produced the stronger association (χ²(4) = 15.795, p = 0.003, Cramér's V = 0.141), and because age is ordinal, a linear-by-linear association test was also applied, which confirmed a graded decline in knowledge with advancing age (M² = 10.61, df = 1, p = 0.001). Gender reached the nominal threshold (χ²(2) = 7.403, p = 0.025, V = 0.136), with men more often in the high knowledge band and women more often in the low band. Both effect sizes are small by conventional criteria. After Bonferroni correction for the 16 tests performed, the age association remains significant while the gender association does not, and the latter should therefore be regarded as suggestive rather than established (Figure 3). 

**Figure 3:**
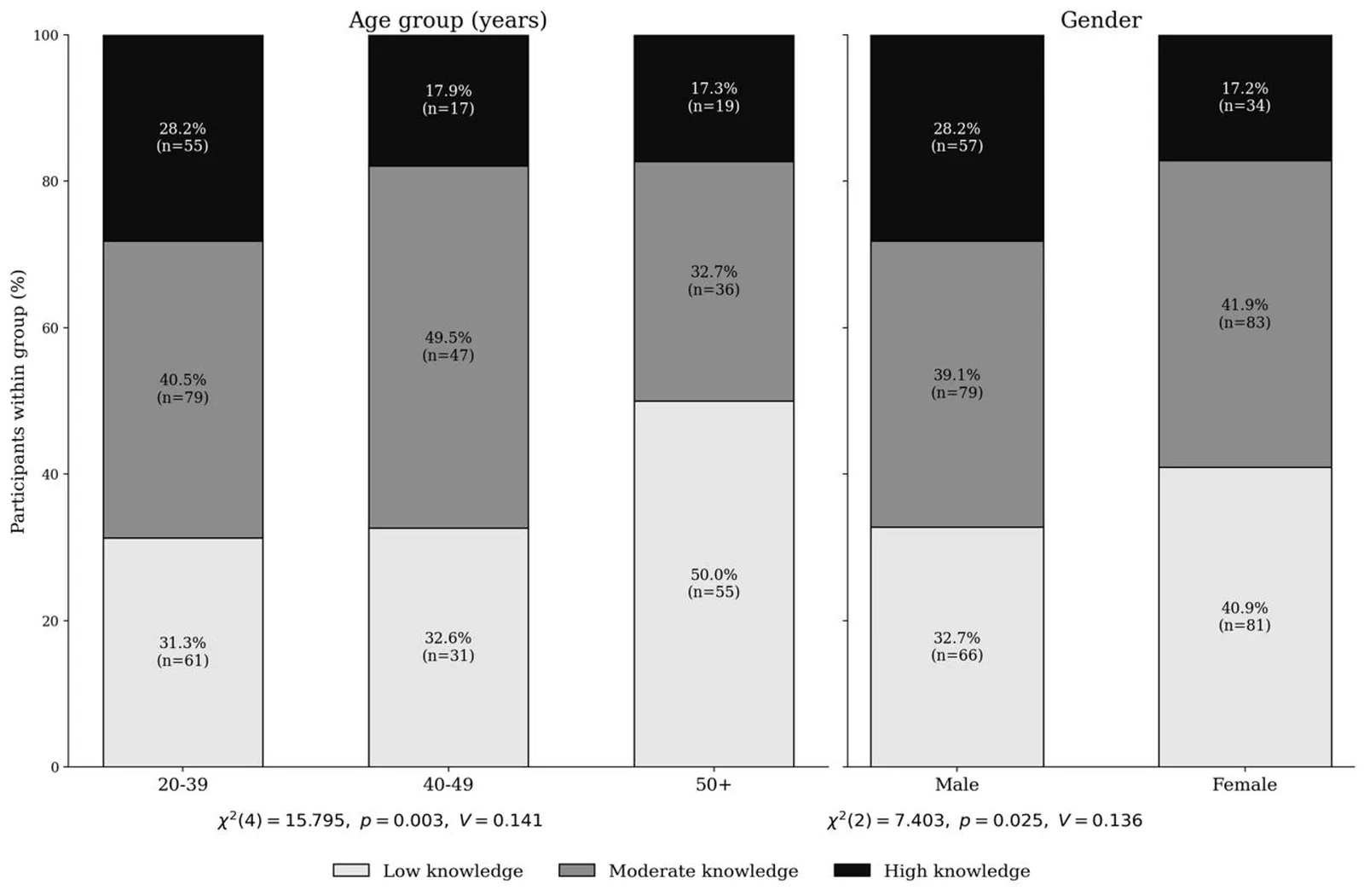
Composite knowledge level by age group and gender (N = 400). Stacked bars show the percentage of participants within each group falling into the low, moderate and high knowledge bands; segments within each bar sum to 100%. The 20-29- and 30-39-year categories were combined before analysis to satisfy Cochran's rule, as described in the Methods. Test statistics, p values and Cramér's V are given beneath each panel. Absolute cell counts are available from the corresponding author.

None of the remaining six variables showed a significant association with knowledge level, and their cross-tabulations are presented in Table 5. Education, socioeconomic status, occupation, place of residence, religion, and PM-JAY beneficiary status were all unrelated to knowledge, with p-values between 0.121 and 0.996 and Cramér's V never exceeding 0.10. The direction of the small differences that did appear was not consistent with a simple gradient of advantage. The highest proportion in the high knowledge band occurred among higher secondary, but the second highest among participants with no formal schooling, and the group least often in the high band was the upper and upper-middle socioeconomic stratum (Table 5).

**Table 5:** Association between composite knowledge level and sociodemographic variables not shown in Figure 3 (N = 400) Cross-tabulation of composite knowledge level against the six sociodemographic variables that were not statistically significant. Age and gender, the two significant associations, are presented in Figure 3 and are not repeated here. Percentages are row percentages within each category. The single Jain respondent was combined with the "Other" religion category and the upper and upper-middle socioeconomic classes were merged, both to satisfy Cochran's rule; the three participants unsure of their PM-JAY status were excluded from that test only (n = 397). No cell in any test had an expected count below 5. df, degrees of freedom; V, Cramér's V; PM-JAY, Pradhan Mantri Jan Arogya Yojana.

Variable	Category	Low, n (%)	Moderate, n (%)	High, n (%)	χ²	df	p value	V
Education	No formal / other (n = 136)	46 (33.8)	59 (43.4)	31 (22.8)	4.854	6	0.563	0.078
	Primary (n = 101)	39 (38.6)	38 (37.6)	24 (23.8)				
	Secondary (n = 128)	51 (39.8)	53 (41.4)	24 (18.8)				
	Higher secondary (n = 35)	11 (31.4)	12 (34.3)	12 (34.3)				
Socioeconomic status	Upper / upper-middle (n = 56)	19 (33.9)	30 (53.6)	7 (12.5)	7.399	6	0.286	0.096
	Middle (n = 120)	46 (38.3)	42 (35.0)	32 (26.7)				
	Lower-middle (n = 146)	51 (34.9)	60 (41.1)	35 (24.0)				
	Lower (n = 78)	31 (39.7)	30 (38.5)	17 (21.8)				
Occupation	Employed (n = 103)	35 (34.0)	45 (43.7)	23 (22.3)	1.264	8	0.996	0.04
	Student (n = 88)	31 (35.2)	35 (39.8)	22 (25.0)				
	Unemployed (n = 82)	32 (39.0)	32 (39.0)	18 (22.0)				
	Self-employed (n = 64)	24 (37.5)	25 (39.1)	15 (23.4)				
	Homemaker (n = 63)	25 (39.7)	25 (39.7)	13 (20.6)				
Residence	Urban (n = 195)	69 (35.4)	71 (36.4)	55 (28.2)	7.302	4	0.121	0.096
	Rural (n = 158)	61 (38.6)	68 (43.0)	29 (18.4)				
	Semi-urban (n = 47)	17 (36.2)	23 (48.9)	7 (14.9)				
Religion	Hindu (n = 164)	58 (35.4)	66 (40.2)	40 (24.4)	4.273	6	0.64	0.073
	Muslim (n = 124)	53 (42.7)	47 (37.9)	24 (19.4)				
	Christian (n = 54)	19 (35.2)	24 (44.4)	11 (20.4)				
	Other (n = 58)	17 (29.3)	25 (43.1)	16 (27.6)				
PM-JAY beneficiary	Yes (n = 173)	62 (35.8)	67 (38.7)	44 (25.4)	1.358	2	0.507	0.058
	No (n = 224)	84 (37.5)	94 (42.0)	46 (20.5)				

Perceived barriers

Endorsement of the five individual barrier items is shown in Figure 4. The striking feature is how little the items separated: all five fell within a band roughly four percentage points wide, so no single obstacle dominated, and participants were, in the main, reporting several simultaneously. Concerns about privacy and possible misuse of health data were endorsed most often, marginally ahead of technical authentication difficulties and limited device or internet access, while the need for staff assistance ranked lowest and still applied to about three-quarters of the sample. The prominence of privacy concerns sits awkwardly beside the high level of expressed trust in data security recorded in Table 3, a tension considered further in the Discussion (Figure 4).

**Figure 4:**
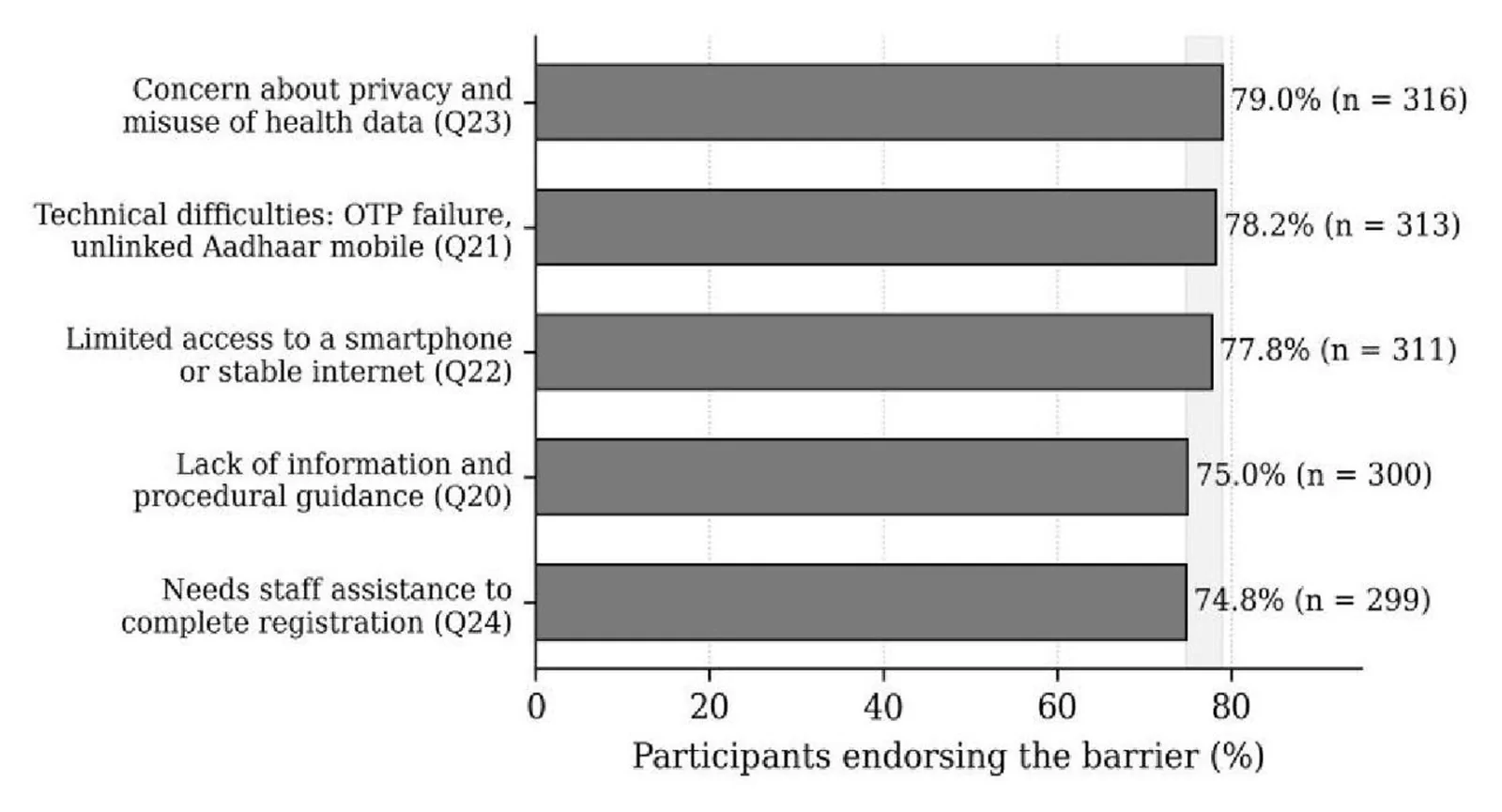
Endorsement of perceived barriers to ABHA use Bars show the percentage of participants answering affirmatively to each barrier item, ordered from most to least frequently endorsed. The shaded band marks the narrow range within which all five items fall. Values give the percentage and the corresponding number of participants. OTP, one-time password; ABHA, Ayushman Bharat Health Account.

Composite barrier level showed no association with any sociodemographic variable. Figure 5 displays the proportion reporting high barriers across every subgroup examined, together with the overall figure for reference. The distribution is essentially flat: no subgroup deviated from the overall value by more than about eleven percentage points, and none of the eight chi-square tests approached significance (all p ≥ 0.164, all Cramér's V ≤ 0.113). The largest test statistic was for education (χ²(3) = 5.109, p = 0.164, V = 0.113), followed by religion (χ²(3) = 3.983, p = 0.263, V = 0.100); the remaining six variables produced p values of 0.415 or above, with PM-JAY beneficiary status showing no difference whatsoever (χ²(1) = 0.000, p = 1.000). This uniformity is a substantive finding rather than an absence of one, and its interpretation is taken up in the discussion. A consolidated summary of all 16 hypothesis tests is provided in Table 6.

**Figure 5:**
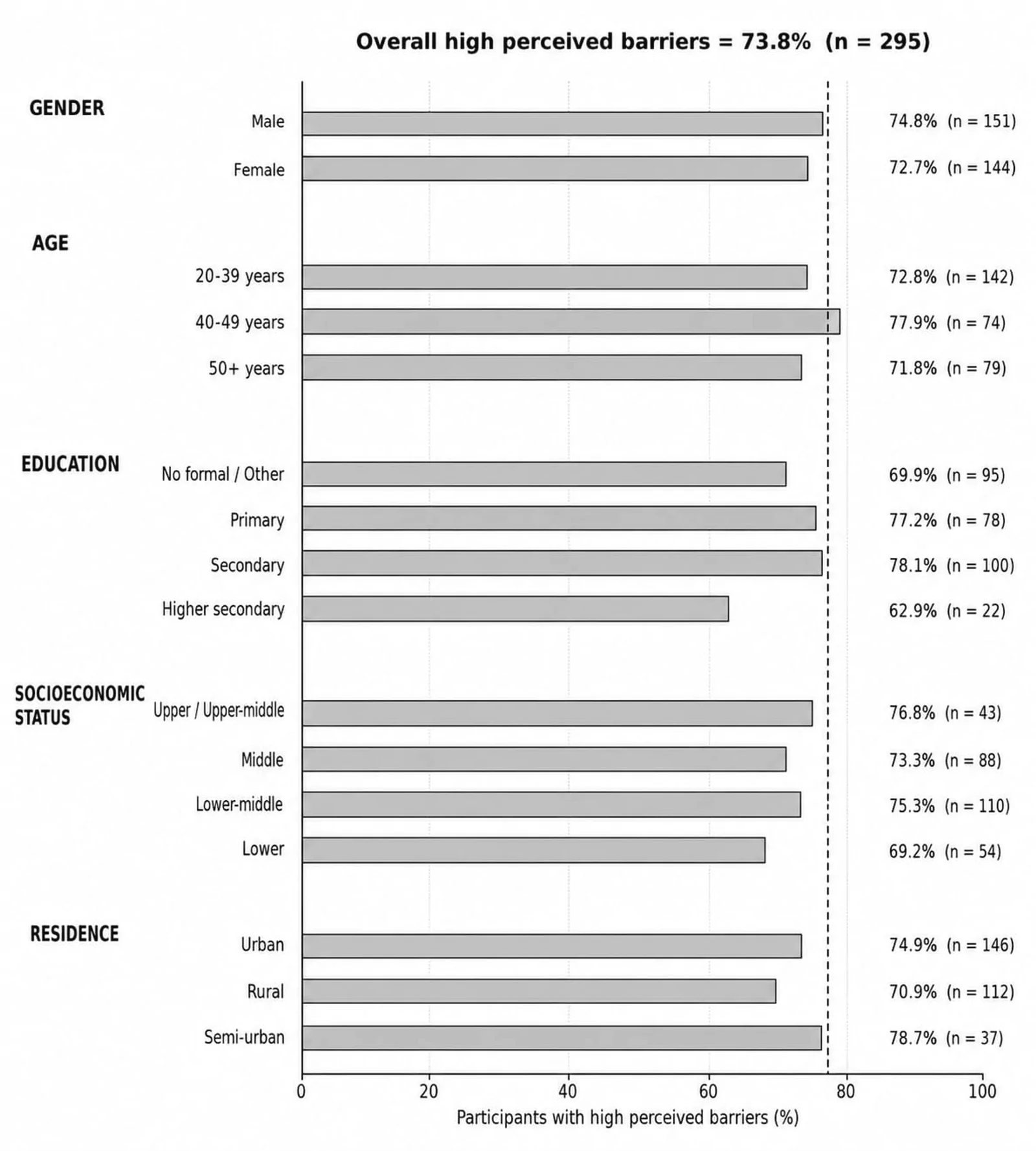
Sociodemographic variation in perceived barriers to ABHA adoption Proportion of participants reporting high perceived barriers, by sociodemographic subgroup (N = 400). Bars show the percentage within each subgroup classified as having high perceived barriers (composite score ≥75% of the maximum). The dashed vertical line marks the overall proportion across the whole sample. Occupation, religion and PM-JAY status are omitted from the display for legibility; their test statistics appear in Table 5. Age categories 20-29 and 30-39 were combined, and the upper and upper-middle socioeconomic classes were merged, to satisfy Cochran's rule. ABHA, Ayushman Bharat Health Account; PM-JAY, Pradhan Mantri Jan Arogya Yojana

**Table 6:** Summary of Pearson chi-square tests for composite knowledge and perceived-barrier levels Consolidated results of all 16 Pearson chi-square tests. Knowledge level was analysed across three bands; barrier level was dichotomised as high versus low or moderate because only 12 participants fell into the low band. All tests are based on N = 400 except those for PM-JAY beneficiary status (n = 397, excluding three participants unsure of their status). No test violated Cochran's rule after the category collapses described in the Methods. * Significant at the nominal threshold of p < 0.05. ‡ Also significant after Bonferroni correction for 16 tests (p < 0.003125); the gender association does not survive this correction. A linear-by-linear association test for the ordinal age variable against knowledge level gave M² = 10.61, df = 1, p = 0.001. df, degrees of freedom; V, Cramér's V; PM-JAY, Pradhan Mantri Jan Arogya Yojana

Exposure	Composite knowledge level				Perceived-barrier level (high vs low/moderate)			
	χ²	df	p value	V	χ²	df	p value	V
Age group	15.795	4	0.003*‡	0.141	1.142	2	0.565	0.053
Gender	7.403	2	0.025*	0.136	0.12	1	0.729	0.017
Education	4.854	6	0.563	0.078	5.109	3	0.164	0.113
Socioeconomic status	7.399	6	0.286	0.096	1.291	3	0.731	0.057
Occupation	1.264	8	0.996	0.04	0.874	4	0.928	0.047
Residence	7.302	4	0.121	0.096	1.397	2	0.497	0.059
Religion	4.273	6	0.64	0.073	3.983	3	0.263	0.1
PM-JAY beneficiary	1.358	2	0.507	0.058	0	1	1	0

## Discussion

This cross-sectional survey of 400 OPD patients at a tertiary hospital in Ranchi found a substantial gap between awareness of ABHA and reported account creation. Fewer than half had heard of ABHA; the mean knowledge score was 56.5% of the maximum, and only 6.0% reported having created an account. Regarding instrument design, items Q4 and Q8 shared near-identical statement wording but intentionally targeted distinct dimensions of health digital literacy: Q8 captured superficial recognition (awareness), whereas Q4 captured procedural comprehension (knowledge). Distinguishing between simple recognition and actionable procedural understanding is essential, as our findings demonstrate that basic awareness of registration routes (Q8: 55.2%) does not automatically translate into functional knowledge (Q4: 16.0%). The largest drop in the enrolment pathway occurred between general awareness and knowledge of the online/app registration route. These findings point to a specific procedural information gap rather than uniformly low interest in ABHA.

Endorsement of Q8 (55.2%) exceeded the proportion of participants who reported prior awareness of the ABHA (Q1: 46.8%). This discrepancy is attributable to the administration of the questionnaire without skip logic, wherein all participants completed every knowledge item regardless of their baseline awareness in Q1. Because Q8 was presented as a declarative statement (“Do you know that...”), participants unacquainted with ABHA by name likely endorsed a plausible assertion upon hearing it--a pattern indicative of acquiescence bias in interviewer-administered surveys. Additionally, familiarity with other digital government portals and mobile applications may have led participants to infer similar operational mechanisms for a national digital health ID. This trend was observed across multiple items, where endorsement rates for specific statements exceeded baseline unprompted awareness. Consequently, item-level and composite knowledge scores represent upper-bound estimates, with Q1 remaining the primary indicator of baseline recognition. Overall, these findings underscore that active, actionable knowledge required for self-registration remains lower than raw score performance suggests.

The registration figure recorded here is consistent with what other Indian studies have found once enrolment is measured rather than assumed. Among underprivileged populations in Bhopal and Raisen districts, registration remained in the low single digits, with absent Aadhaar and unlinked mobile numbers acting as hard constraints [9]. An operational audit at a tertiary centre in Eastern India attributed integration failure principally to the absence of a smartphone, poor signal reception, and patient refusal, with refusal alone accounting for over a fifth of failures [10]. The contrast with the Chandigarh study is instructive: uptake there reached 78%, but registration was actively facilitated on site during the mission's pilot phase, and even in that sample only 35.6% had heard of ABDM beforehand [7]. Where enrolment is assisted, it happens; where it depends on the patient finding their own way, it largely does not. A hospital survey in urban Bengaluru, reporting ABDM awareness of just 8% among outpatients who were otherwise comfortable with government digital services, points to the same conclusion from the opposite direction [8].

The pattern of knowledge across demographic groups was narrower than expected. Age was the only variable to survive correction for multiple testing, and the linear-by-linear trend test confirmed a graded decline rather than an isolated outlier group, with participants aged 50 and above roughly half as likely as the youngest group to reach the high knowledge band and half of them falling into the low band. This is the familiar generational gradient in digital familiarity, and it identifies older outpatients as the group least able to navigate self-registration unaided. Gender reached the nominal threshold, but not the corrected one, and its effect size was small. Given that this is a single-centre convenience sample and that sixteen tests were performed, the honest reading is that the gender difference is a signal worth following in a larger study rather than a finding on which policy should turn. Reporting it as established would overstate what these data support, although its direction is consistent with work showing that self-directed digital pathways tend to widen rather than narrow existing gaps unless assistance is deliberately built in [11].

What did not show an association is equally informative. Education, socioeconomic status, occupation, residence, religion and PM-JAY status were all unrelated to knowledge, with effect sizes below 0.10 throughout. The small differences that did appear sometimes ran against expectation, most notably the upper and upper-middle stratum being the least likely to reach the high knowledge band. Two explanations deserve consideration. The first is simply that this study was not powered to detect small differences within subgroups, and the confidence one can place in a null result at these effect sizes is limited. The second is more substantive: patients attending a busy public tertiary hospital share a common information environment of registration counters, posters, staff announcements and conversation in the queue, which may homogenise basic familiarity in a way that formal education does not. Studies reporting education as a positive correlate of ABHA awareness have generally sampled community populations rather than a single hospital queue [9], so the settings are not directly comparable and the divergence is not necessarily a contradiction.

The barrier results were the most uniform in the study, and this uniformity is the finding rather than a null result to be set aside. Not one of the eight sociodemographic variables was associated with barrier level; effect sizes were negligible throughout, and the proportion reporting high barriers varied only modestly across every subgroup examined. At item level, the five barriers clustered within a four-point range. Two implications follow. First, the obstacles patients described are properties of the enrolment process rather than characteristics of particular patients, which argues for a universal facilitation model within the hospital rather than one targeted at demographic subgroups. Second, the apparent contradiction between widespread privacy concern and equally widespread expressed trust in data security is worth taking seriously rather than explaining away. General confidence in the system appears to coexist with specific unease about one's own records, a combination also seen in the Chandigarh cohort, where confidence in government data security predicted uptake even as security worries remained common [7]. Abstract trust, in other words, does not neutralise concrete hesitation at the point of enrolment.

Set against these barriers is a genuinely receptive patient population. Roughly three-quarters agreed that digital records would improve their care and would encourage relatives to register, and support for a hospital helpdesk or kiosk was the strongest attitude recorded anywhere in the survey. Low registration in this setting therefore reflects a supply-side and process failure rather than patient resistance, and that distinction matters because the remedies for scepticism and the remedies for friction are not the same. The efficiency case for closing the gap is already documented: a time-series comparison of online ABHA-based registration against conventional outpatient registration at an Indian tertiary hospital found substantial reductions in registration time [17], and a multi-hospital study of OPD attendees in Agra linked ABHA adoption to higher digital health literacy and better patient satisfaction [18]. At RIMS, with daily OPD attendance of 2,100 to 3,000, those gains remain almost entirely unrealised [13].

Three specific interventions follow directly from these data. First, assisted enrolment at the point of care addresses the largest single knowledge deficit, because telling patients that an online route exists is of little use when barriers to using it are near-universal; an on-site desk bypasses the question altogether. Second, Aadhaar and mobile number seeding should be treated as a prerequisite step rather than a downstream troubleshooting task, given how frequently authentication difficulty was reported. Third, communication needs to correct a specific misconception rather than raise awareness in general. With fewer than half the participants able to distinguish ABHA from PM-JAY, and with a majority of this sample reporting that they were not PM-JAY beneficiaries, patients who know they fall outside the insurance scheme may reasonably conclude that ABHA is not for them. Messaging that separates the two schemes explicitly is likely to be more useful than messaging that promotes ABHA in the abstract. Older patients should be prioritised in the design of assisted pathways, with attention to women as a secondary consideration pending confirmation of that association in a larger sample.

Limitations

This study has several limitations that should be considered when interpreting the findings. First, the cross-sectional design precludes establishing causal relationships, and the single-centre setting at a public tertiary care teaching hospital limits direct generalizability to primary healthcare centres, rural populations, or other states. Furthermore, convenience sampling in outpatient waiting lounges may have introduced selection bias by over-representing stable patients able to endure extended wait times, while under-sampling acutely unwell patients, time-pressed individuals, or those who departed before being approached. Additionally, the study did not measure participants' prior exposure to external ABHA promotional campaigns or media drives, which represents an unmeasured potential confounder for baseline awareness.

Second, outcome measurement relied on self-reported registration status without direct digital portal verification, introducing potential recall uncertainty, while expressed attitudes may have been influenced by social desirability bias. Regarding instrument properties, although the knowledge tool underwent content validation, it lacked formal test-retest reliability testing. Moreover, the five-item combined perceived difficulty/support scale exhibited modest internal consistency (α = 0.26), reflecting the broad, multi-dimensional spectrum of individual, technical, and institutional obstacles evaluated; nevertheless, sensitivity checks demonstrated 90% concordance in participant risk classifications, and individual item-level frequencies were reported alongside composite scores to ensure transparency.

Third, analytical granularity was constrained by data processing choices: age was recorded only in broad categorical brackets rather than as a continuous variable, and certain sociodemographic categories were collapsed to meet statistical cell-count assumptions, reducing measurement detail. Because analyses were unadjusted (bivariate), residual confounding cannot be ruled out, and the sample size was not powered to detect small effect sizes. Finally, given that multiple statistical comparisons were executed, only the association between age and composite knowledge remained robust following Bonferroni correction, whereas other secondary comparisons should be interpreted as exploratory.

## Conclusions

In this single-centre convenience sample of 400 outpatients at a government tertiary care teaching hospital in Ranchi, knowledge of ABHA was limited (mean 56.5% of the maximum score) and self-reported registration was low (6.0%, with a further 10.0% unsure). The largest fall between successive stages was from having heard of ABHA (46.8%) to knowing that an account can be created online or through an app (16.0%), although a similarly worded item (Q8) was endorsed more often (55.2%). Older patients had lower knowledge, and perceived barriers were widespread across subgroups; other sociodemographic associations were not significant, which, given the limited power, is inconclusive. These findings are descriptive and self-reported, and they do not show why registration was low. Hospital-based assisted registration, particularly for older patients, is a hypothesis that merits prospective evaluation in multicentre studies with portal-verified registration data.

## Appendices

Appendix 1 

Reporting follows the Strengthening the Reporting of Observational Studies in Epidemiology (STROBE) statement for cross-sectional studies (Table 7).

**Table 7:** Strengthening the Reporting of Observational Studies in Epidemiology (STROBE) statement for cross-sectional studies STROBE, Strengthening the Reporting of Observational Studies in Epidemiology. Items 1 to 12 relate to the title, abstract, introduction and methods; items 13 to 17 to results; items 18 to 22 to discussion and other information.

Item	Recommendation topic	STROBE recommendation	Location in manuscript
1	Title and abstract	(a) Study design indicated in the title. (b) Structured abstract summarising background, methods, results and conclusions.	Title; Abstract
2	Background/rationale	Scientific background and rationale for the investigation reported.	Introduction, paragraphs 1 to 5
3	Objectives	Specific objectives stated.	Introduction, final paragraph
4	Study design	Key elements of study design presented early in the paper.	Abstract (Methods); Materials and Methods, Study design and setting
5	Setting	Setting, locations, and relevant dates including periods of recruitment and data collection.	Materials and Methods, Study design and setting; Data collection (May to July 2026)
6	Participants	Eligibility criteria and sources and methods of selection of participants.	Materials and Methods, Study population and eligibility criteria; Sample size and sampling technique
7	Variables	All outcomes, exposures, predictors and potential confounders defined, with diagnostic criteria.	Materials and Methods, Study instrument and domain structure; Scoring and classification
8	Data sources / measurement	Sources of data and details of methods of assessment for each variable of interest.	Materials and Methods, Study instrument and domain structure; Data collection
9	Bias	Efforts to address potential sources of bias described.	Materials and Methods, Study instrument and domain structure (expert content validation, pilot testing on 40 excluded patients); Discussion, Limitations
10	Study size	Explanation of how the study size was arrived at.	Materials and Methods, Sample size and sampling technique (n = 196, 218 after non-response allowance, expanded to 400)
11	Quantitative variables	Explanation of how quantitative variables were handled, and groupings described with rationale.	Materials and Methods, Scoring and classification (including rationale for the revised scoring rule and identical banding of both composites)
12	Statistical methods	(a) All statistical methods described, including those used to control for confounding. (b) Methods for subgroup examination. (c) Handling of missing data. (d) Sampling strategy accounted for. (e) Sensitivity analyses.	Materials and Methods, Statistical analysis. (a) Unadjusted analyses only; no confounder control, acknowledged in Limitations. (b) Eight sociodemographic subgroups; category collapses to satisfy Cochran's rule described. (c) No missing data; all 400 participants completed every item. (d) Convenience sample; no weighting applied. (e) Sensitivity analysis under the earlier scoring rule reported in Scoring and classification.
13	Participants	(a) Numbers of individuals at each stage. (b) Reasons for non-participation. (c) Flow diagram if applicable.	Results, Participant characteristics. All 400 recruited participants completed the interview; no flow diagram required.
14	Descriptive data	(a) Characteristics of study participants and information on exposures and potential confounders. (b) Number of participants with missing data for each variable.	Results, Participant characteristics; Table 1. No missing data for any variable.
15	Outcome data	Numbers of outcome events or summary measures reported.	Results, The awareness-to-action gap; Item-level knowledge; Attitudes and facilitators; Composite knowledge and barrier levels. Figures 1, 2 and 4; Tables 2 and 3.
16	Main results	(a) Unadjusted estimates and, where applicable, confounder-adjusted estimates with precision. (b) Category boundaries reported when continuous variables were categorised. (c) Translation of relative risk into absolute risk where meaningful.	Results, Sociodemographic associations with knowledge; Perceived barriers. Figures 3 and 5; Tables 4 and 5. Unadjusted estimates with Cramer's V as the precision measure; category boundaries in Materials and Methods, Scoring and classification. Not applicable to (c).
17	Other analyses	Other analyses performed, such as subgroup and interaction analyses and sensitivity analyses.	Results, Table 5 (16 subgroup tests with Bonferroni threshold stated); linear-by-linear trend test for age; sensitivity analysis under the earlier scoring rule described in Materials and Methods. No interaction analyses performed.
18	Key results	Key results summarised with reference to study objectives.	Discussion, paragraph 1; Conclusions
19	Limitations	Limitations discussed, taking into account sources of potential bias or imprecision, and both direction and magnitude of any potential bias.	Discussion, Limitations (two paragraphs covering sampling, self-report, social desirability, multiplicity, category collapsing and power)
20	Interpretation	Cautious overall interpretation of results considering objectives, limitations, multiplicity of analyses, results from similar studies, and other relevant evidence.	Discussion, paragraphs 2 to 7; Conclusions
21	Generalisability	Generalisability (external validity) of the study results discussed.	Discussion, Limitations; Conclusions, final paragraph
22	Funding	Source of funding and the role of the funders for the present study and, if applicable, for the original study on which the present article is based.	Additional Information, Disclosures. No funding was received.

Appendix 2 

Data were collected using a self-designed and structured, pre-tested bilingual (English and Hindi) questionnaire (Table 8).

**Table 8:** Questionnaire used for the study

Study Information & Consent		
Question / Field (English / हिंदी)		
Study Title: To assess the awareness of patients regarding the registration process of the Ayushman Bharat Health Account (ABHA) at a tertiary healthcare facility in Jharkhand अध्ययन शीर्षक: झारखंड के एक तृतीयक स्वास्थ्य संस्थान में आयुष्मान भारत स्वास्थ्य खाता (ABHA) पंजीकरण के प्रति मरीजों की जागरूकता का आकलन। Purpose: This study evaluates patients' awareness and understanding of the registration process of the Ayushman Bharat Health Account (ABHA) and identifies knowledge gaps that may guide interventions to improve ABHA registration and use. उद्देश्य: यह अध्ययन आयुष्मान भारत हेल्थ अकाउंट (ABHA) के पंजीकरण प्रक्रिया के प्रति रोगियों की जागरूकता का आकलन करता है तथा पंजीकरण एवं उपयोग बढ़ाने हेतु ज्ञान की कमियों की पहचान करता है। Participation: Your participation is voluntary. You may withdraw anytime without consequences. The survey takes about 10-15 minutes. भागीदारी: आपकी सहभागिता स्वैच्छिक है और आप कभी भी बिना किसी दंड के हट सकते हैं। सर्वेक्षण में लगभग 10-15 मिनट लगेंगे। Confidentiality: Responses are anonymous and confidential, used only for research. गोपनीयता: आपके जवाब गोपनीय और गुमनाम रहेंगे, केवल शोध के लिए उपयोग होंगे। Risks/Benefits: No risks; your input may improve ABHA use in healthcare. जोखिम/लाभ: कोई जोखिम नहीं है; आपका सुझाव स्वास्थ्य सेवाओं में ABHA के उपयोग में सुधार करने में सहायक हो सकता है।		
		Consent: By signing below, you confirm that you are a patient/participant aged 20 years or older, have understood the study information, and agree to participate voluntarily. सहमति: नीचे हस्ताक्षर करके, आप पुष्टि करते हैं कि आपकी आयु 20 वर्ष या उससे अधिक है, आपने अध्ययन की जानकारी समझ ली है और स्वेच्छा से भाग लेने के लिए सहमत हैं।	Signature/ हस्ताक्षर	
		Demographic Details		
		#	Question / Field (English / हिंदी)	Options (English / हिंदी)
		1	Participant ID प्रतिभागी आईडी	(to be filled) (भरा जाना है)
2	Age Group आयु समूह	20–29 years / 30–39 years / 40–49 years / 50 years and above 20–29 वर्ष / 30–39 वर्ष / 40–49 वर्ष / 50 वर्ष और अधिक
3	Gender आपका लिंग	Male / Female पुरुष / महिला
4	Education शैक्षणिक योग्यता	Higher Secondary / Secondary / Primary / No formal education / Other उच्च माध्यमिक / माध्यमिक / प्राथमिक / कोई औपचारिक शिक्षा नहीं / अन्य
5	Religion आपका धर्म	Hindu / Muslim / Christian / Sikh / Buddhist / Jain / Other (specify) / Prefer not to say हिन्दू / मुस्लिम / ईसाई / सिख / बौद्ध / जैन / अन्य (बताएं) / बताना नहीं चाहते
6	Socio-economic Status सामाजिक-आर्थिक स्थिति	Upper class / Upper-middle class / Middle class / Lower-middle class / Lower class उच्च वर्ग / उच्च-मध्यम वर्ग / मध्यम वर्ग / निम्न-मध्यम वर्ग / निम्न वर्ग
7	Occupation आप क्या काम करते हैं	Student / Homemaker / Employed (specify) / Self-employed / Unemployed छात्र/छात्रा / घर का काम / नौकरी (बताएं) / अपना काम/व्यवसाय / बेरोज़गार
8	Place of Residence निवास स्थान	Rural / Semi-urban / Urban ग्रामीण / अर्ध-शहरी / शहरी
9	Are you a beneficiary under Ayushman Bharat – PMJAY? क्या आपके पास आयुष्मान भारत (PMJAY) कार्ड है?	Yes / No / Not sure हाँ / नहीं / पता नहीं
Awareness & Knowledge about ABHA Registration Process		
#	Question / Field (English / हिंदी)	Options (English / हिंदी)
1	Do you know about ABHA (Ayushman Bharat Health Account)? मैंने आयुष्मान भारत हेल्थ अकाउंट (ABHA) के बारे में सुना है।	Yes / No / Not sure हाँ / नहीं / पता नहीं
2	Do you know ABHA is primarily used for digital storage of health records? ABHA मुख्य रूप से स्वास्थ्य रिकॉर्ड को डिजिटल रूप से स्टोर करने के लिए उपयोग किया जाता है।	Yes / No / Not sure हाँ / नहीं / पता नहीं
3	Do you know that an ABHA number can be generated using Aadhaar Card? मुझे पता है कि ABHA नंबर बनाने के लिए आधार कार्ड का उपयोग किया जा सकता है।	Yes / No / Not sure हाँ / नहीं / पता नहीं
4	Do you know that ABHA registration can be done through Mobile App/Online Portal? मुझे पता है कि ABHA पंजीकरण मोबाइल ऐप/ऑनलाइन पोर्टल के माध्यम से किया जा सकता है।	Yes / No / Not sure हाँ / नहीं / पता नहीं
5	Do you know that ABHA registration is free of cost? मुझे पता है कि ABHA पंजीकरण निःशुल्क है।	Yes / No / Not sure हाँ / नहीं / पता नहीं
6	Do you know that ABHA helps link past health records digitally for future access? मुझे पता है कि ABHA पुराने स्वास्थ्य रिकॉर्ड को डिजिटल रूप से लिंक करने में मदद करता है ताकि भविष्य में आसानी से एक्सेस किया जा सके।	Yes / No / Not sure हाँ / नहीं / पता नहीं
7	Have you personally created an ABHA number? मैंने व्यक्तिगत रूप से ABHA नंबर बनाया है।	Yes / No / Not sure हाँ / नहीं / पता नहीं
8	Do you know that an ABHA number can be registered at an Online Portal/App? मुझे पता है कि ABHA नंबर ऑनलाइन पोर्टल/ऐप में बनाया जा सकता है।	Yes / No / Not sure हाँ / नहीं / पता नहीं
9	Do you know that an internet connection may be required for ABHA registration? मुझे पता है कि ABHA पंजीकरण के लिए इंटरनेट कनेक्शन की आवश्यकता हो सकती है।	Yes / No / Not sure हाँ / नहीं / पता नहीं
10	I know that an ABHA number can be linked to a mobile number. मुझे पता है कि ABHA नंबर को मोबाइल नंबर से लिंक किया जा सकता है।	Yes / No / Not sure हाँ / नहीं / पता नहीं
11	I know Aadhaar Card is mandatory for ABHA registration. मुझे पता है कि ABHA पंजीकरण के लिए आधार कार्ड अनिवार्य है।	Yes / No / Not sure हाँ / नहीं / पता नहीं
12	I know that once an ABHA number is created, it can be used in multiple hospitals. मुझे पता है कि एक बार ABHA नंबर बनने के बाद, वही नंबर कई अस्पतालों में इस्तेमाल किया जा सकता है।	Yes / No / Not sure हाँ / नहीं / पता नहीं
13	I know that ABHA can be used to access lab reports, prescriptions, and medical history online. मुझे पता है कि ABHA का उपयोग करके लैब रिपोर्ट, प्रिस्क्रिप्शन और मेडिकल हिस्ट्री ऑनलाइन देखी जा सकती है।	Yes / No / Not sure हाँ / नहीं / पता नहीं
14	I think that ABHA is different from the Ayushman Bharat Health Insurance Card (PMJAY). मुझे लगता है कि ABHA आयुष्मान भारत हेल्थ इंश्योरेंस कार्ड (PMJAY) से अलग है।	Yes / No / Not sure हाँ / नहीं / पता नहीं
Attitude and Perceived Barriers toward ABHA Registration		
#	Question / Field (English / हिंदी)	Options (English / हिंदी)
15	Do you believe ABHA is a useful initiative for improving healthcare services? मुझे लगता है कि ABHA से इलाज की सुविधा बेहतर होती है।	Yes / No / Not sure हाँ / नहीं / पता नहीं
16	Are you willing to encourage your family and friends to register for ABHA? मैं अपने परिवार और दोस्तों को ABHA बनवाने के लिए कहूँगा/कहूँगी।	Yes / No / Not sure हाँ / नहीं / पता नहीं
17	Do you think a dedicated helpdesk or registration kiosk at the hospital would increase your likelihood of registering for ABHA? मुझे लगता है कि अस्पताल में एक अलग सहायता केंद्र (हेल्पडेस्क) या पंजीकरण कियोस्क होने से मेरे ABHA बनवाने की संभावना बढ़ जाएगी।	Yes / No / Not sure हाँ / नहीं / पता नहीं
18	Do you think ABHA would reduce paperwork and waiting time during hospital visits? मुझे लगता है कि ABHA से अस्पताल में कागजी कार्रवाई और प्रतीक्षा समय कम हो जाएगा।	Yes / No / Not sure हाँ / नहीं / पता नहीं
19	Do you trust that your personal health data under ABHA will remain private and secure? मुझे भरोसा है कि ABHA में मेरी स्वास्थ्य जानकारी सुरक्षित रहती है।	Yes / No / Not sure हाँ / नहीं / पता नहीं
20	Do you think lack of awareness is a major barrier to ABHA registration? ABHA के बारे में जानकारी की कमी एक बड़ी समस्या है।	Yes / No / Not sure हाँ / नहीं / पता नहीं
21	Do technical difficulties act as a barrier to ABHA registration? तकनीकी समस्या (मोबाइल, OTP, नेटवर्क आदि) ABHA बनवाने में परेशानी करती है।	Yes / No / Not sure हाँ / नहीं / पता नहीं
22	Do you think limited access to the internet or smartphones affects ABHA registration? इंटरनेट या स्मार्टफोन न होने से ABHA बनवाने में दिक्कत होती है।	Yes / No / Not sure हाँ / नहीं / पता नहीं
23	Do you fear that data misuse discourages people from registering for ABHA? लोगों को डर रहता है कि उनकी जानकारी का गलत इस्तेमाल हो सकता है।	Yes / No / Not sure हाँ / नहीं / पता नहीं
24	Do you think awareness drives and staff assistance would improve ABHA registration among patients? क्या आपको लगता है कि जागरूकता कार्यक्रम और अस्पताल के स्टाफ की सहायता से ABHA बनवाना आसान हो सकता है?	Yes / No / Not sure हाँ / नहीं / पता नहीं
